# Special Issue: Probabilistic Mechanical Fatigue and Fracture of Materials

**DOI:** 10.3390/ma13214901

**Published:** 2020-10-31

**Authors:** Miguel Muñiz-Calvente, Alfonso Fernández-Canteli

**Affiliations:** Department of Construction and Manufacturing Engineering, University of Oviedo, 33203 Gijón, Spain; afc@uniovi.es

**Keywords:** fatigue, fracture mechanics, phenomenological models, failure criterion, generalized driving force, elastic and plastic materials, probabilistic life prediction, environmental assisted fatigue

## Abstract

When designing structural and mechanical components, general structural integrity criteria must be met in order to ensure a valid performance according to its designed function, that is, supporting loads or resisting any kind of action causing stress and strains to the material without catastrophic failure. For these reasons, the development of solutions to manage the test conditions, failure mechanism, damage evolution, component functionalities and loading types should be implemented. The aim of this Special Issue “Probabilistic Mechanical Fatigue and Fracture of Materials” is to contribute to updating current and future state-of-the-art methodologies that promote an objective material characterization and the development of advanced damage models that ensure a feasible transferability from the experimental results to the design of real components. This is imbricated in some probabilistic background related to theoretical and applied fracture and fatigue theories, and advanced numerical models applied to some real application examples.

## 1. Introduction

The engineering design of structural and mechanical components is based on the fulfillment of general structural integrity criteria. This ensures that the component will satisfactorily perform its designed function, supporting loads or resisting any kind of action causing stress and strains to the material without catastrophic failure. Consequently, during recent decades a large number of studies have been performed to allow fatigue and fracture to be reliably predicted. Nevertheless, the diversity of test conditions, failure mechanisms, damage evolution, component functionalities and loading types, as well as the specificity of material properties and the difficulty of achieving unambiguous material characterization, have impeded the discovery of a satisfactory solution to all failure questions.

The main purpose of this Special Issue is to contribute to updating current and future state-of-the-art methodologies that facilitate an objective material characterization and the development of advanced damage models that guarantee the transfer from experimental results to the design of real components. This being encompassed in some probabilistic background related to innovative experimental technologies, theoretical and applied facture and fatigue theories, advanced numerical models and examples of real applications related to advanced materials.

This Special Issue is composed by two main topics: fracture and fatigue. The fracture topics addressed in the papers of this Special Issue are summarized in this editorial, emphasizing their main contributions to the state-of-the-art methodologies. After this, the topics related to the fatigue problem are treated in the second part. Finally, as a complementary but interrelated topic, the viscoelastic characterization is also enriched with the development of statistical models.

## 2. Fracture Topics

Regarding fracture topics, this Special Issue addresses two important and ongoing engineering issues, such as the interrelation between fracture results of different experimental procedures in soil-cement materials and the probabilistic fracture characterization of notched components and specimens.

The fracture characterization usually involves various heterogeneous experimental procedures with different types of loading in the specimens. One of the critical points, when several kinds of experiments are required in the characterization process, is the interrelation of the experimental results from one type of test to another. This is the case for soil-cement usually employed in road construction. On the one hand, it is common to use the well-known uniaxial compressive strength (UCS) test at seven days, included in national and international standards. However, the alternative flexural strength (FS) allows the mechanical behaviour of the soil-cement to be better defined in the long-term. To this aim, Linares-Unamunzaga et al. [1] proposes analytical solutions to the problem of interrelation of the experimental results within these two types of experimental procedures. As a result, different regression models are developed, providing simple solutions (linear multiple regression) to more complicated scenarios by considering physical magnitudes (density, etc.) based on the Cobb–Douglas model production function. These proposals were corroborated in 63 prismatic samples of soil-cement manufactured in situ.

Furthermore, the presence of notches and notched-type defects exemplifies one of the common causes of fracture in real structures and mechanical components. These structural details, such as joints, rivets and holes, are known to act as stress concentrators around the notch tip, promoting premature failures of specimens or components under service conditions. Form the early efforts of calculating the influence of the stress distribution on the stress intensity factor, to present times, several researchers have proposed different methodologies for fracture characterization of notched specimens, with almost all of them being deterministic. However, the significant and non-negligible scatter associated with fracture results must be addressed by means of suitable probabilistic approaches, such as the proposal by Muñiz-Calvente et al. [2]. This proposed methodology, based on the so-called generalized local model (GLM), allows for the primary failure cumulative distribution function (PFCDF) to be derived as a material property, that is, being independent of the test type, load conditions and specimen geometry. As a result, a joint evaluation of experimental fracture results coming from different notch types can now be performed, since a unique PFCDF arises as the representative one for the material. The authors corroborate the proposed methodology with an experimental campaign on EPOLAM2025 epoxy resin.

## 3. Fatigue Topics

Regarding fatigue topics, this Special Issue deals with an extensive list of current ongoing issues, such as the surface effect on the fatigue strength, material selection methods in real applications related to dental materials, experimental techniques to be used in the fatigue crack growth, numerical studies of the plastic crack tip opening displacement (CTOD), development of statistical methods to assess the fatigue models (Bayesian techniques in the S-N field and the effect of a varying ultimate number of cycles in the runout) and, finally, the use of novel spectral methods in the frequency-domain approach to fatigue lifetime prediction.

As it is well known, the surface quality has a noticeable effect on the fatigue strength of components, as occurs with the use of titanium and its alloys because of their low surface hardness and a high friction coefficient. Thus, the development of surface modification techniques to improve the fatigue properties related to the surface effect represents a task to be addressed by researchers and engineers, such as heat treatments, surface coating with films and thermo-chemical treatments. Among the latter treatments, the gas nitriding technique has been widely used, providing a poor final surface finish with long duration of time. Therefore, the alternative plasma nitriding was preferred instead, as in the work by de Castro et al. [3] with a titanium Ti-6Al-4V alloy. The comparative analysis suggested by the authors provides interesting results: (a) the plasma nitrided specimens exhibit higher low-cycle fatigue strength compared with similar behaviour for high cycles with those non-nitrided; (b) the fracture surfaces in the nitrided specimens show raised regions, characteristic of plastic deformation prior to fracture, while in the untreated specimens large regions with planar propagation are found instead, related to the final rupture.

The material selection in pipe industry for gas transportation represents a critical stage in the engineering design, since the duration of these infrastructures is usually around 30 years. Due to the gas interaction with steel materials, these pipes are often insulated with mastic and film protective coatings trying to avoid the hydrogenation process. The identification of the stages in this process has already been performed, being significantly dependent on the particular climatic zones. For this reason, the purpose of the work of Hutsaylyuk et al. [4] is to establish general regularities and patterns of these structures guiding the prevention and maintenance programs after long-term operation over 30–39 years, aiming at reliable structural integrity predictions. Within their findings, the hydrogenation process was accompanied by microplastic deformation and nucleation of micropores due to static deformation, with both zones of ductile separation and individual regions of micro-spallation found in the fracture surface. However, the steels used in this work show a sufficiently high level of mechanical properties are required for further safe exploitation in terms of durability and cracking resistance.

Aigner et al. [5] address the surface effect on fatigue lifetime problems from a theoretical perspective, focused on the so-called statistical size effect. The distribution of the defect sizes plays a central role in the probabilistic analysis of fatigue fracture initiating defect sizes in a high-stress volume, together with the scale-effect within two different volumes (specimen A: lower, specimen B: higher), which represents a novel approach since current existing methodologies are focused on the statistical size effect due to natural defects. As a result, the statistical size effect is related to a noticeable decrease in fatigue strength in terms of specimen geometry B compared with A, the estimation of the theoretical distribution of the defects was corroborated with a minor deviation for a probability of occurrence of 50% and thus, the defect size could be considered as acting as an equivalent crack size for the fatigue assessment with respect to its extensions for short crack growth in the Kitagawa–Takahashi diagram.

The selection of suitable restorative materials in dental applications also represents an important task to be addressed for dentists and researchers. The real service conditions of these materials in tooth structure are restrictive, not only because of the complex systems of mechanical forces acting simultaneously in the mastication process, but also due to the acid media surrounding them. For this reason, the development of suitable selection methods of restorative materials satisfies these concurrent requirements. Current methodologies are only focused on the mechanical properties of the selected material, ignoring the condition related to the acid media in which the tooth structure uses to work. To this purpose, Colombo et al. [6] extend the micro-hardness techniques to materials after being exposed to acidic drink, allowing both effects to be taken into account in the selection of the restorative material. In particular, the authors applied the proposed technique to four different typical dental materials: a force-absorbing hybrid ceramic block, a resin nano ceramic, a nanohybrid composite and a zirconia-reinforced lithium silicate glass ceramic. Within them, the ceramics were found to show greater mechanical characteristics than resin components, besides being less affected by the acidic solution.

From an experimental viewpoint, the study of the fatigue crack propagation is still an ongoing engineering problem. Some of the currently used methodologies reported a mechanism through fracture morphology combined with undamaged microstructural features. Nevertheless, these methods observe cracks indirectly without the behaviour of material near the crack so that the interpretation of fracture morphology requires experienced researchers. To this aim, some advanced methods like the electron backscattered diffraction (EBSD) and the scanning electron microscope (SEM) were applied and the research could be focused on the crack behaviour and the deformation around it. Liu et al. [7] applied the EBSD technique for studying the crack propagation stage in a friction stir welded plated of 7N01 Al-Zn-Mg alloy. It follows that the resistance for fatigue crack propagation in the stir zone is lower than that in the base metal, with a tendency for intergranular growth with a smaller plastic zone being identified. The strain in this stir zone is also concentrated with lattice distortion due to the strengthening effect of grain boundaries, while this deformation region in the base metal is large and continuous. Additionally, different factors affecting the fatigue crack were observed simultaneously, such as second phase particles, recrystallized grains and grain boundaries, all of them contributing to a local incompatible strain.

Due to the multidependent character of the fatigue crack growth process with respect to a large variety of variables and factors (variability of the material, loading, geometry, temperature, etc.), current methods resort to the use of safety factors as a conservative approach, or by means of stochastic analysis, such as the proposal of Prates et al. [8]. The authors perform a sensitivity analysis of the fatigue crack growth process based on numerical simulations, with respect to different factors (influence of material parameters and loading variability) in order to quantify their effects, in terms of the variability of the plastic CTOD. Among the most relevant findings, the Young’s modulus, the initial yield stress and the maximum applied force were found to be the most influential parameters and the predictable variability in the plastic CTOD range can be represented with right-skewed distributions.

The development of mathematical and statistical models for fatigue lifetime prediction must be accompanied with suitable assessment methods to quantify the associated uncertainties. This is the case of the work by Castillo et al. [9], where the well-known Castillo–Canteli probabilistic model [10] is enhanced with the Bayesian technique to calculate the percentiles of the percentiles in the S-N field. As a result, the confidence intervals of the original percentile curves of probability of failure can now be interpreted as their confidence intervals, providing with more reliable predictions for fatigue failure. Additionally, the typical question about the suitable number of tests to carry out in an experimental S-N fatigue program is required to achieve reliable or confident results to be used in component design.

Another pending goal in the design of fatigue experimental campaigns is the assessment of the limit number of cycles in the very high cycle region where the runouts are usually assumed to be established at 10^6^–10^7^ cycles, which is an important topic addressed by Geilen et al. [11]. In this work, the authors propose a new method denoted artificial censoring experiment (ACE), where an original fatigue model fitted with real data, such as the well-known bi-linear model, is compared with the resulting model if artificial censored data are simulated beyond the original ultimate number of cycles. As a result, if both original and artificial models provide similar lifetime predictions in terms of the 50% quantile at different load levels, the original model could be considered as valid for extrapolation beyond the ultimate number of cycles. This proposed methodology is illustrated with an experimental campaign on compression springs manufactured from VDSiCr, where the ultimate number of cycles is a controversial decision since tests on springs of large diameters beyond 10^9^ cycles would last one year.

Finally, it is important to mention that, although fatigue lifetime prediction is traditionally performed in the time-domain from load histories in the form of stress or strain, fatigue failure can be also addressed in the frecuency-domain. As well kwon, when random vibration fatigue is involved and the load history cannot be easily defined, the frequency-domain or spectral methods are preferable, based on more computationally efficient procedures. Nevertheless, these methods are rarely applied in notched conditions, or when the amplitude is above the yield’s stress and the fatigue lifetime prediction is limited to elastic deformations. For this reason, Böhm et al. [12] proposes a novel algorithm to extend the spectral methods for considering elastoplastic deformations and notched conditions. The proposed algorithm is successfully exemplified with an experimental campaign on 0H18N9 steel in uniaxial bending loading.

## 4. Complementary Topics

Finally, the Special Issue also addresses the development of statistical models in a different context from those aforementioned, all of them related to the fatigue and fracture of metals. The viscoelastic materials are nowadays of great interest in mechanical and structural applications, encompassing a wide variety of different kinds of materials, such as polymers, tissues and woods. The main characteristic is their time and frequency dependent properties, which influence the mechanical characterization methods to be used. Indeed, due to this long-term behaviour, over several decades in time or frequency, the experimental methods are impelled to be tested within a limited time interval. For this reason, alternative methods are required in order to predict the time-dependent properties beyond the limits of the experimental window (see Ferry [13]). One of the procedures widely used consists of testing at various temperatures and applying the well-known time–temperature superposition (TTS) principle. However, there are some disadvantages to this and inconsistencies arise/appear in its application to real data. To this aim, Álvarez-Vázquez et al. [14] propose an alternative interpretation of the TTS principle, modelling the time evolution of the relaxation curves at different temperatures as statistical distributions pertaining to the extended normal distribution and Gumbel distribution. Indeed, the development of suitable methodologies for viscoelastic characterization represents the first stage in further reliable predictions for the facture of this kind of components.
